# A potential role for the adrenal gland in autism

**DOI:** 10.1038/s41598-021-97266-8

**Published:** 2021-09-07

**Authors:** Felwah S. Al-Zaid, Abdel Fattah A. Alhader, Laila Y. Al-Ayadhi

**Affiliations:** 1 Department of Physiology, College of Medicine, King Saud University, P O Box 2925, Riyadh, 11461 Kingdom of Saudi Arabia; 2 Autism Research and Treatment Center, College of Medicine, King Saud University, Riyadh, Kingdom of Saudi Arabia; 3 Department of Physiology, College of Medicine, Jordan University of Science and Technology, Irbid, Jordan

**Keywords:** Neurology, Neurological disorders

## Abstract

Androgens have been implicated in autism pathophysiology as recently, prenatal exposure to elevated androgens has been proposed as risk factor. However, published data on postnatal sex hormone levels in autistic children are controversial and the source of prenatal androgen exposure in autism remains unknown. Therefore, this study investigated postnatal sex hormone levels and dehydroepiandrosterone (DHEA) to shed light on a potential role for the adrenal gland in autism pathophysiology. A case-control study investigating estradiol (E2), DHEA, follicle-stimulating hormone (FSH) and luteinizing hormone (LH) levels was conducted with 31 Saudi males with autism and 28 healthy, age-matched boys plasma. Moreover, correlation analysis with measured hormones and previously measured total testosterone (TT) and free testosterone (FT) in the same group of autism was conducted. DHEA was significantly higher (*p* < 0.05) in the autism group compared to controls. DHEA positively correlated with previously measured TT (r = + 0.79, *p* < 0.001) and FT (r = + 0.72, *p* < 0.001) levels in the same autism group. FSH levels were also significantly higher in the autism group than in the control group (*p* < 0.01). To the best of our knowledge, this is the first study to report a strong positive correlation between TT, FT and DHEA, suggesting an adrenal source for elevated androgen levels.

## Introduction

Sex hormones have recently garnered the interest of researchers for their roles in autism. This interest is not only due to the striking male-to-female ratio in autism but also due to the dramatic effect of steroids on brain development and behavior. The empathizing–systemizing (E–S) theory of autism^1^ supports the clinical presentation of the disease and its strong association with the male sex. This theory proposes that females have stronger empathizing tendencies, which reflects their ability to identify with the mental status of others and to respond with appropriate emotions. On the other hand, systemizing, which is stronger in males than in females, reflects the drive to analyze a system in terms of a set of governing rules^2^. A further extension of the E–S theory of autism is the extreme male brain (EMB) theory, which proposes that females have stronger empathizing tendencies and males have stronger systemizing tendencies; thus, autism can be considered an extreme of the normal male profile^3^.

Human studies have shown that prenatal exposure to high levels of fetal testosterone results in the masculinization of the brain^4^. This phenomenon was first observed in girls with congenital adrenal hyperplasia (CAH). These females are prenatally exposed to high levels of testosterone and subsequently display autistic behaviors^5^. This observation led to the hypothesis that one cause of autism is prenatal exposure to high levels of fetal testosterone.

Baron–Cohen and colleagues were recently the first to report a direct link between elevated testosterone levels in the womb and the development of autism^6^; however, the source of these elevated androgen levels remains unclear.

Numerous studies have been conducted to investigate postnatal testosterone levels in autistic children. Although some studies demonstrated that children with autism have significantly elevated androgen levels^7^, other studies, such as those of Tordjmann et al*.*^8^ and Lutchmaya et al*.*^9^, did not find significant differences between testosterone levels in autistic children and normal children. Moreover, postpubertal testosterone levels in autistic males have been shown to be lower than those in normal males^10^.

To investigate the potential role of steroid hormones in the pathophysiology of autism in Saudi boys, this study measured the serum levels of 17-β estradiol (E_2_) and dehydroepiandrosterone (DHEA) as well as the levels of the gonadotropins follicle-stimulating hormone (FSH) and luteinizing hormone (LH) in autism and control subjects. We recently reported the TT, FT and sex hormone-binding globulin (SHBG) levels in the same group of patients and controls included in this study^11^. These data are crucial for the completion of the steroid hormone profiles and the determination of their correlations with DHEA and other measured hormones.

## Results

**Figure 1:**
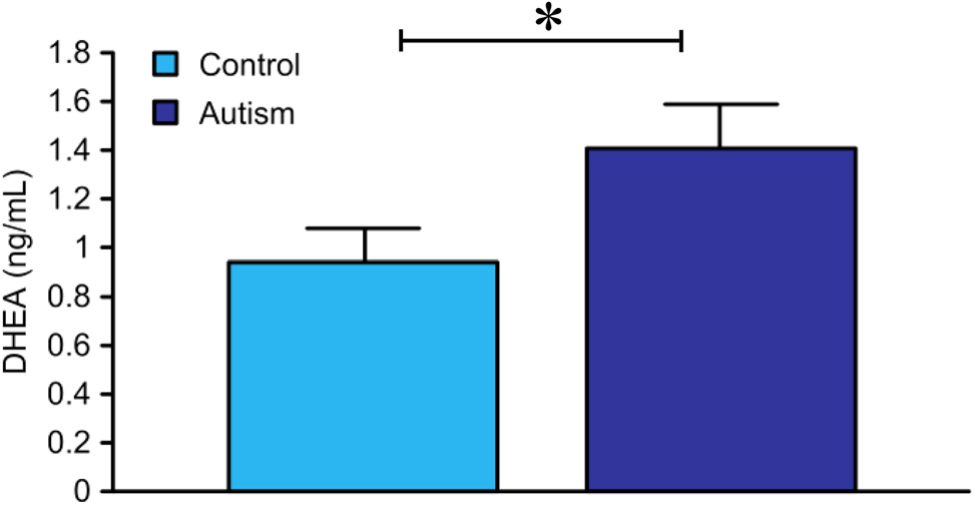
DHEA in both the control and autism groups (* = designates statistical significance compared to controls).

The means of the anthropometric data for the autism and control groups are presented in Table 1. A significant difference was observed in the average body weights of the two groups. The mean levels of the previously measured TT, FT, SHBG and the currently measured E_2_, DHEA, FSH, LH for both the control and autism groups are shown in Table 2. A significant increase in TT and FT was previously reported and the current study showed significantly higher DHEA (Fig. 1) and FSH levels in the autism group than in the age- and sex-matched control group. Correlation analysis between different variables are presented in Table 3, which shows a strong positive correlation between DHEA measured in the current study and both TT and FT which were shown to be correlated together in our previous study^11^ in the same group of autism. Table 3 also shows strong positive correlations between both TT and FT with different physical parameters. The outputs of the logistic regression modeling revealed that only DHEA, FT, and FSH remained significant until the final step of modeling factors associated with autism status. Inclusion of weight in the model, that adjusted for the confounding effect, lead to disappearance of the significant association between Leptin and autism which was detected previously in the bivariate analysis. The results of the logistic regression modeling are in Table 4.

**Table 1:** Clinical characteristics of normal controls and subjects with autism.

Parameter	Control group (n = 28)	Autism group (n = 31)	*p* Value
Wt (kg)	19.3 ± 0.8	22.7 ± 1.4	0.05*
Ht (cm)	112 ± 1.4	117.9 ± 2.6	0.06
Waist circ. (cm)	52.1 ± 1.1	55.7 ± 1.6	0.06
Hip circ. (cm)	59.4 ± 1.1	62.6 ± 1.8	0.14
Waist/Hip	0.88 ± 0.01	0.89 ± 0.01	0.22
Age (months)	65.3 ± 2.8	67.19 ± 3.73	0.68
BMI (kg/m^2^)	15.3 ± 0.4	15.9 ± 0.5	0.28
Head circ. (cm)	51.2 ± 1.2	51.7 ± 2.3	0.29

*Abbreviations*: N, number; Wt, weight; Ht, height; BMI, body mass index; Waist circ., waist circumference; Hip circ., hip circumference; Head circ., head circumference. Data are presented as means ± standard deviation.*p ≤ 0.05 is considered statistically significant.

**Table 2:** Sex hormones and SHBG levels in the control and autism groups.

Hormone	Control group	Autism group	*p* Value	df	T value
TT (ng/dL)	13.1 ± 6.7	20.1 ± 17.4	0.05*	57	2.068
FT (pg/dL)	26.8 ± 22.9	90.8 ± 77.9	0.001*	57	4.367
SHBG (nmol/L)	101.8 ± 30.1	106 ± 42.4	0.66	57	0.443
DHEA (ng/mL)	0.94 ± 0.7	1.4 ± 1	0.05*	57	2.044
E_2_ (pg/mL)	3.3 ± 3.3	4.2 ± 2.4	0.22	57	1.102
FSH (mIU/ml)	0.49 ± 0.3	0.88 ± 0.6	0.01*	57	3.077
LH (mIU/ml)	0.87 ± 0.4	0.97 ± 0.4	0.37	57	0.887

TT, FT and SHBG were reported in our previously published study^11^.

Data are presented as the mean ± standard deviation.

*p ≤ 0.05 is considered statistically significant.

**Table 3:** Correlation between different variables in the autism group.

Variables	Pearson correlation coefficient	*p* Value
TT & DHEA	+ 0.79	0.001*
TT & FT	+ 0.70	0.001*
TT & 2nd:4th ratio	+ 0.36	0.05*
TT & age	+ 0.44	0.05*
TT & waist circumference	+ 0.37	0.05*
TT & hip circumference	+ 0.37	0.05*
FT& DHEA	+ 0.72	0.001*
FT & waist circumference	+ 0.59	0.001*
FT & hip circumference	+ 0.52	0.01*
FT & height	+ 0.49	0.01*
FT & weight	+ 0.54	0.01*
FT & BMI	+ 0.38	0.05*
Leptin & weight	+ 0.67	0.001*
Leptin & BMI	+ 0.56	0.001*

*p ≤ 0.05 is considered statistically significant.

**Table 4:** The estimates of significant associations between autism and hormones concentration using logistic regression modeling.

Independent variables	Odds ratio	Confidence interval 95%		*p* Value
		Lower limit	Upper limit	
(Intercept)	0.13	0.03	0.53	0.001*
DHEA	2.01	1.01	4.00	0.045*
FSH	7.49	1.70	32.99	0.008*

*p ≤ 0.05 is considered statistically significant.

## Discussion

The results of this study did not reveal significant differences between the anthropometric measurements of the autistic and control subjects, except for body weight. The average body weight of the autistic children was 18% higher (*p* ≤ 0.05) than that of the control children, which is consistent with the results of several other reports that have observed a higher average body weight in autistic children^12–14^. This observation can be explained in part by factors related to physical activity and eating behaviors, as children with autism have lower levels of physical activity because their social impairments can limit their participation in structured physical games^15^. Moreover, children with autism have been reported to have unusual eating habits that are generally described as overly selective. Specifically, Schreck and Williams^16^ found that autistic children are highly selective for energy-dense foods (e.g., peanut butter, cake, and hot dogs). Therefore, these differences in physical activity and eating behaviors likely explain the higher average body weight that has been observed in autistic children. Interestingly, we previously found a strong positive correlation between weight and leptin^11^ in the same group of autistic children (r = + 0.67, *p* ≤ 0.001) as well as between leptin and BMI (r = + 0.56, *p* ≤ 0.001). Moreover, significant positive correlations between previously measured TT and both waist and hip circumference were observed (r = + 0.37, *p* ≤ 0.05; r = 0.37, *p* ≤ 0.05, respectively), which is consistent with previously observed correlations^17^. A wide range of significant positive correlations between FT and physical parameters have also been observed. These included correlations with waist circumference (r = + 59, *p* ≤ 0.001), hip circumference (r = + 0.52, *p* ≤ 0.01), height (r = + 0.49, *p* ≤ 0.01), weight (r = + 0.54 *p* ≤ 0.01) and BMI (r = + 0.40, *p* ≤ 0.05). The positive correlations of the previously measured FT and TT with the above mentioned physical parameters indicated a physical anabolic effect of androgens in autism. A relationship that is further supported by the additional observation of a strong positive correlation between the previously measured leptin and FT (r = + 0.42, *p* ≤ 0.01). The close association between plasma levels of leptin and testosterone has been reported by Paolisso et al.^17^ and highlighted the significance of this correlation in clinical practice^10,18^. These findings suggest that the anti-androgen therapy suggested by Geier et al.^7^ could influence leptin and subsequently weight in individuals with autism.

Studies of postnatal androgen levels in autistic children have produced conflicting results. Some investigations have reported high androgen levels in autistic children^6^, while other studies have reported no significant differences between the androgen levels in autistic children and those of their age-matched controls^8,9^. Moreover, post-pubertal androgen levels have been found to be lower in autistic children than in typically developing children^10^. However, the role of androgens in Saudi autistic children has never been assessed. We previously demonstrated significant elevations in TT, which was 53% higher in the autistic children than in the control children (*p* ≤ 0.05), and FT, which was 238% higher in the autistic children than in the control children (*p* ≤ 0.001) with no significant differences in SHBG levels (*p* = 0.66) between the autism group and the control group^11^. In that study, the significant between-group difference in FT levels exceeded the difference in TT levels by an order of magnitude, which was explained by the lack of a concomitant difference in SHBG levels. Both TT and FT levels were positively and strongly correlated with each other (r = + 70, *p* ≤ 0.001), and they also showed positive and strong correlations with DHEA levels measured in the current study (r = + 79, *p* ≤ 0.001, and r = + 72, *p* ≤ 0.001, respectively). In the current study, DHEA levels were significantly higher (50%, *p* ≤ 0.05) in autistic children than in control children, suggesting that the source of the elevated androgens is the zona reticularis of the adrenal gland, as it is the main source of circulating DHEA^19^. A significant elevation in these androgens compared with laboratory reference values has been reported in American autistic children in Geier’s study, which reported 158% higher TT levels, 214% higher FT levels and 192% higher DHEA levels in autistic children^7^. On the other hand, Croonenberghs and colleagues reported that the testosterone levels of children with autism (12–18 years of age) were significantly lower than those of their age-matched controls. These results do not conflict with the results of the present study for two reasons: first, the age group included in Croonenberghs’ study was post puberty; second, the lower testosterone level observed in autistic children in this age group can be explained by greater negative feedback at the hypothalamic level. In human, the preoptic region in the hypothalamic nucleus is androgen sensitive and is 2.5 times larger in males than in females^20^. The prenatal growth of this region is strongly determined by the presence of testosterone. Too much testosterone early in life can influence androgen receptors and their sensitivity in this region. As children age, namely, when they reach puberty, this could cause negative feedback, resulting in lower testosterone concentrations^10^. On the other hand, Tordjmann et al*.*^8^ and Lutchmaya et al*.*^9^ reported nonsignificant differences in testosterone levels of autistic children and age-matched controls. However, it is important to note that Tordjmann reported remarkable heterogeneity in the group of subjects involved in the study, which included pre- and postpubertal children.

The significantly higher levels of androgens and the significantly lower 2nd-to-4th digit ratio (which is a ratio calculated by dividing the length of the 2nd hand digit (index finger) by the length of the 4th hand digit (ring finger) reported^21^ in the same group of Saudi autistic children included in this study relative to those of age-matched control subjects are consistent with the fetal androgen and EMB theories of autism^2^. In addition, high testosterone levels in mice model of autism have been associated with moodiness, low attachment, and low sociability in the pre-pubertal stage, which are commonly observed characteristics of autistic children^22^.

The influence of genetics on disrupted androgen regulation observed in autistic children was reported in a recent study that linked polymorphism in the androgen receptor gene (SRD5A2) to autism in Slovak children^23^. More recently, the discovery of dysregulation of the RORA gene in autism and its influence on the aromatase enzyme, a key regulator of the sex hormone biosynthesis pathway, provides a potentially broader explanation for the link between autism and hyperandrogenism^24^. Another explanation for the elevated androgen levels in autistic children was suggested by Geier and colleagues, who reported a significant abnormality in the DHEA synthesis pathway in autistic children. Normally, DHEA can either be converted into the storage molecule dehydroepiandrosterone sulfate (DHEA-S) or into testosterone or estrogen. In patients with autism, a decrease in trans-sulfuration metabolites was observed, resulting in a marked decrease in DHEA-S production and a subsequent increase in testosterone^7^.

The present study also showed that the FSH levels of Saudi autistic children were significantly higher (80%) than those of their age-matched controls. This result was not consistent with the reported results from two other studies. Iwata and colleagues recently found no significant difference between the FSH levels of an autism group and an age-matched control group^25^. Moreover, Geier’s study reported a significant decrease in the FSH levels in autistic subjects compared with laboratory reference values^7^. Circulating FSH levels in childhood and early puberty exhibit sexual dimorphism; girls have higher levels of circulating FSH than boys do because of the higher levels of the negative regulator inhibin-B in boys^26,27^. The significantly elevated levels of FSH in Saudi autistic children could be due to decreased levels of inhibin-B and the resulting lack of negative regulation of FSH levels. Moreover, inhibin-B was found to be negatively regulated by testosterone via direct androgenic action in a study that showed that testosterone treatment in pre-pubertal boys was associated with a significant decrease in inhibin-B levels, which could, in turn, lead to an increase in FSH levels^28^. This would be consistent with our previously reported significant elevation of testosterone^11^ in the same group of autistic children included in the current study. This proposed mechanism of FSH elevation in autistic subjects requires further study of inhibin-B levels in autistic children.

In the present study, when FT introduced to the regression model, it leads to many problems in the model such as inversion of the association between DHEA and autism or what is known as collider effect. The collider factor is a common effect of both independent, in this case DHEA, and dependent factor, in this case autism, which lead to inversion of the estimate calculated by a regression model. The inclusion of FT in the model changed OR for the relation between DHEA and autism to be 0.11 which indicates a lower level of DHEA in autism children when compared to that in controls. Thus, FT variable was removed from the model and final model only included DHEA and FSH as predictors for autism.

Another possible explanation for these findings is the association of leptin and ghrelin with reproductive hormones. This association was reported in our previous study^11^ that investigated the relationship between ghrelin, leptin and reproductive hormones; it revealed a negative correlation between ghrelin and FSH levels and a positive correlation between leptin and FSH levels^29^. Using the present cohort of Saudi children, we recently reported significantly lower ghrelin levels and significantly higher leptin levels in autistic children compared with their age-matched controls^11^. Moreover, leptin regulates the hypothalamus–pituitary–gonad axis at both the central and gonadal levels^30^. Leptin stimulates LH and FSH in pituitary gonadotrophs^31^. Higher FSH levels and lack of significantly higher LH levels in the Saudi autistic children compared to their age-matched controls can be explained by the suppression of LH release by their significantly elevated testosterone levels; this negative feedback regulation was reported in normal men and pubertal boys after testosterone infusion^32,33^. Another possible explanation is orexin deficiency in autistic children, as orexin deficiencies are known to reduce the release of LH without affecting the release of FSH. Orexin has been reported to play a role in modulating GnRH burst frequencies, as high-frequency bursts enhance LH secretion and low-frequency bursts promote FSH release^34^. Although the role of orexin in autism has not yet been addressed, orexin has been implicated in psychiatric disorders^35^. Additionally, orexin is known to act in central nervous system (CNS) brain regions, such as the hippocampus, which is known to be affected in autism^36^. Moreover, leptin, which was reported in our previous study to be present at significantly higher levels in the autism group than in the control group^11^, both are the same groups included in the current study, is known to inhibit the release of orexin^37^. On the other hand, ghrelin, which was found to be present at significantly lower levels in Saudi autistic children compared to their age-matched controls in our previous study^11^ that was conducted on same group of autism included in the current study, is known to stimulate the release of orexin^38,39^; thus, orexin levels are likely to be low in autistic children.

Our findings in the current study and previous work suggest a wide range of hormonal dysregulation in boys with autism, in which we propose androgens as the pace-maker for this hormonal dysregulation. Furthermore, we suggest adrenals as the source for the elevated androgens in boys with autism. However, limitations to be addressed as this study was conducted on male gender autism children and their age and sex matched controls due to lake of female subjects.

## Conclusion

Our results showed a significant elevation in DHEA and FSH levels in boys with autism compared to their age- and sex-matched controls. This is the first study to report a strong positive correlation between the previously reported elevated levels of TT and FT and the DHEA levels measured in the current study in the same group of boys with autism, suggesting an adrenal source for the elevated androgens. Considering androgens’ capacity to exert organizational effects on the developing human brain, our findings raise a hypothesis that the adrenal gland could be the pathological origin for autism, which is worth further investigation and represents a potential target for early therapeutic interventions.

## Methods

### Subject selection

This case-control study was conducted with 59 male children, 31 of whom had classic-onset autism (the autism group) and 28 of whom were age- and sex-matched healthy children (the control group). The study participants ranged from 3 to 8 years of age (mean ± SD = 5.59 ± 2.26 years) and were prepubertal (Tanner stage 1). The boys with autism were recruited from the Autism Research and Treatment Center at the College of Medicine of King Saud University, Riyadh, Saudi Arabia. The study was limited to the male sex because of the lack of female subjects in the Autism Research and Treatment Center. The subjects in this study fulfilled the criteria for the diagnosis of autism according to the 4^th^ edition of the Diagnostic and Statistical Manual of Mental Disorders (DSM-IV)^40^. The Autism Diagnostic Observation Schedule (ADOS) and the Childhood Autism Rating Scale (CARS) were used to make the diagnosis of autism according to the DSM-IV. The patients included in this study had no associated neurological diseases, such as cerebral palsy or tuberous sclerosis, or metabolic disorders, such as phenylketonuria and were not intellectually disabled. The experimental protocols as well as informed written consent obtained from the patients’ parents are in accordance with the guidelines of the Institutional Review Board (IRB) of the College of Medicine at King Saud University, Riyadh, Saudi Arabia and, also, approved by the IRB (approval # E-10-341).

### Anthropometric measurements

The height and weight of all of the participants were measured using an electronic scale. The occipitofrontal head circumference, waist circumference and hip circumference were measured with a measuring tape. The body mass index (BMI; weight (kg)/height (m^2^)) and waist-to-hip ratio were also calculated.

### Biochemical assays

Five milliliters of venous blood was collected in a plain plastic tube between 8:00 am and 9:00 am after the participants had completed an overnight fast. The collected blood was used to measure the DHEA, E2, FSH and LH levels. The samples were centrifuged for 10 min at 3500 rpm, and the serum samples were immediately separated and stored at − 80 °C until they were assayed without further freeze/thaw cycles.

For the hormonal analyses, enzyme-linked immunosorbent assay (ELISA) kits (DIAsource ImmunoAssays S.A., Rue de l’Industrie, B-1400 Nivelles, Belgium) were used to measure DHEA (KAPDB490), E_2_ (KAP0621), FSH (KAPD1288) and LH (KAPD1289) levels.

### Statistical analysis

The data were analyzed using SPSS PC+ (Version 18.0) software. Independent samples t-tests were used to compare the means between the autism and control groups. The correlations between different variables were determined using Pearson correlation coefficients. The data are presented as the means ± SD, and statistical significance is defined as *p* ≤ 0.05. We used a logistic regression analysis to test the relation between hormone levels and autism. All hormones of interest were included as independent variables in the analysis TT, FT, E2, DHEA, FSH, LH, SHBG, and Leptin; in addition to weight and interaction term between Leptin and weight. Autism status (autistic or not) was the dependent variable.

Interaction term between Leptin and weight was not significant. Thus, we dropped the interaction term and run a logistic regression model with step-wise backward selection using Wald test in SPSS, which automatically drops the non-significant variable from a model.

### Laboratory data

#### Sex hormones and sex hormone-binding globulin

The mean levels of TT, FT, E_2_, DHEA (Fig. 1), FSH, LH and SHBG for the control group and the autism group are presented in Table 2. The correlation analysis between different variables is shown in Table 3.

## Data Availability

Authors declare availability of data with no restrictions.

## References

[1] Baron-Cohen S (2009). Autism: The empathizing–systemizing (E–S) theory. Ann. N. Y. Acad. Sci..

[2] Baron-Cohen S, Knickmeyer RC, Belmonte MK (2005). Sex differences in the brain: Implications for explaining autism. Science.

[3] Schulkin J (2007). Autism and the amygdala: An endocrine hypothesis. Brain Cogn..

[4] Hines M, Constantinescu M, Spencer D (2015). Early androgen exposure and human gender development. Biol. Sex Differ..

[5] Knickmeyer R (2006). Androgens and autistic traits: A study of individuals with congenital adrenal hyperplasia. Horm. Behav..

[6] Baron-Cohen S (2015). Elevated fetal steroidogenic activity in autism. Mol. Psychiatry.

[7] Geier DA, Geier MR (2007). A prospective assessment of androgen levels in patients with autistic spectrum disorders: Biochemical underpinnings and suggested therapies. Neuro Endocrinol. Lett..

[8] Tordjman S (1995). Plasma androgens in autism. J. Autism Dev. Disord..

[9] Lutchmaya S, Baron-Cohen S, Raggatt P, Knickmeyer R, Manning JT (2004). 2nd to 4th digit ratios, fetal testosterone and estradiol. Early Hum. Dev..

[10] Croonenberghs J (2010). Serum testosterone concentration in male autistic youngsters. Neuro Endocrinol. Lett..

[11] Al-Zaid FS, Alhader AA, Al-Ayadhi LY (2014). Altered ghrelin levels in boys with autism: A novel finding associated with hormonal dysregulation. Sci. Rep..

[12] Sugiyama T (1991). A research of obesity in autism. Jpn. J. Dev. Disabil..

[13] Dreyer M, Egan A, Kippes C, Andrews J, May M (2008). Obesity and overweight among patients diagnosed with autism spectrum disorders. Obesity.

[14] Curtin C, Anderson SE, Must A, Bandini L (2010). The prevalence of obesity in children with autism: A secondary data analysis using nationally representative data from the National survey of children's health. BMC Pediatr..

[15] Dziuk MA (2007). Dyspraxia in autism: Association with motor, social, and communicative deficits. Dev. Med. Child Neurol..

[16] Schreck KA, Williams K (2006). Food preferences and factors influencing food selectivity for children with autism spectrum disorders. Res. Dev. Disabil..

[17] Paolisso G, Rizzo MR, Mone CM, Tagliamonte MR, Gambardella A, Riondino M, Carella C, Varricchio M, D'Onofrio F (1998). Plasma sex hormones are significantly associated with plasma leptin concentration in healthy subjects. Clin. Endocrinol..

[18] Utriainen P, Laakso S, Liimatta J, Jaaskelainen J, Voutilainen R (2015). Premature adrenarche—A common condition with variable presentation. Horm. Res. Paediatr..

[19] Auchus RJ, Rainey WE (2004). Adrenarche—Physiology, biochemistry and human disease. Clin. Endocrinol. (Oxf.).

[20] Bao A-M, Swaab DF (2011). Sexual differentiation of the human brain: Relation to gender identity, sexual orientation and neuropsychiatric disorders. Front. Neuroendocrinol..

[21] Al-Zaid FS, Alhader AA, Al-Ayadhi LY (2015). The second to fourth digit ratio (2D:4D) in Saudi boys with autism: A potential screening tool. Early Hum. Dev..

[22] Brodkin ES (2007). BALB/c mice: Low sociability and other phenotypes that may be relevant to autism. Behav. Brain Res..

[23] Schmidtova E, Kelemenova S, Celec P, Ficek A, Ostatnikova D (2010). Polymorphisms in genes involved in testosterone metabolism in Slovak autistic boys. Endocrinologist.

[24] Sarachana T, Xu M, Wu RC, Hu VW (2011). Sex hormones in autism: androgens and estrogens differentially and reciprocally regulate RORA, a novel candidate gene for autism. PLoS ONE.

[25] Iwata K (2011). Investigation of the serum levels of anterior pituitary hormones in male children with autism. Mol. Autism.

[26] Winter JS (1982). Hypothalamic–pituitary function in the fetus and infant. Clin. Endocrinol. Metab..

[27] Lee, P. A. Neuroendocrine maturation and puberty. In *Pediatric and Adolescent Obstetrics and Gynecology* 12–26 (Springer, 1985).

[28] Foster CM, Olton PR, Racine MS, Phillips DJ, Padmanabhan V (2004). Sex differences in FSH-regulatory peptides in pubertal age boys and girls and effects of sex steroid treatment. Hum. Reprod..

[29] El-Eshmawy MM, Abdel Aal IA, El Hawary AK (2010). Association of ghrelin and leptin with reproductive hormones in constitutional delay of growth and puberty. Reprod. Biol. Endocrinol..

[30] Pasquali R (2006). Obesity and androgens: Facts and perspectives. Fertil. Steril..

[31] Yu WH, Kimura M, Walczewska A, Karanth S, McCann SM (1997). Role of leptin in hypothalamic-pituitary function. Proc. Natl. Acad. Sci. U. S. A..

[32] Kletter GB (1991). Naloxone does not reverse the suppressive effects of testosterone infusion on luteinizing hormone secretion in pubertal boys. J. Clin. Endocrinol. Metab..

[33] Kletter GB, Foster CM, Beitins IZ, Marshall JC, Kelch RP (1992). Acute effects of testosterone infusion and naloxone on luteinizing hormone secretion in normal men. J. Clin. Endocrinol. Metab..

[34] Foster CM (1989). Testosterone infusion reduces nocturnal luteinizing hormone pulse frequency in pubertal boys. J. Clin. Endocrinol. Metab..

[35] Marshall JC, Eagleson CA, McCartney CR (2002). Hypothalamic dysfunction. Mol. Cell. Endocrinol..

[36] Borgland SL, Labouèbe G (2010). Orexin/hypocretin in psychiatric disorders: Present state of knowledge and future potential. Neuropsychopharmacology.

[37] DeLong GR (1992). Autism, amnesia, hippocampus, and learning. Neurosci. Biobehav. Rev..

[38] Ohno K, Sakurai T (2008). Orexin neuronal circuitry: Role in the regulation of sleep and wakefulness. Front. Neuroendocrinol..

[39] Toshinai K (2003). Ghrelin-induced food intake is mediated via the orexin pathway. Endocrinology.

[40] American Psychiatric Association (1994). Diagnostic and statistical manual of mental disorders: DSM-IV.

